# State of the Art in Smart Portable, Wearable, Ingestible and Implantable Devices for Health Status Monitoring and Disease Management

**DOI:** 10.3390/s22114228

**Published:** 2022-06-01

**Authors:** Shouvik Mukherjee, Shariq Suleman, Roberto Pilloton, Jagriti Narang, Kirti Rani

**Affiliations:** 1 Department of Biotechnology, School of Chemical and Life Sciences, Jamia Hamdard, Hamdard Nagar, New Delhi 110062, India; shvkmkhrj@gmail.com (S.M.); shariqsuleman07@gmail.com (S.S.); 2 National Research Council (CNR), Department of Chemical Sciences and Materials Technology (DSCTM), Institute of Crystallography (IC), Via Salaria Km. 29.3, Monterotondo, 00185 Rome, Italy; 3 Amity Institute of Biotechnology, Amity University Uttar Pradesh Noida (Uttar Pradesh), Gautam Buddha Nagar 201303, India; krsharma@amity.edu

**Keywords:** smart devices, wearable devices, portable devices, implantable devices, ingestible devices

## Abstract

Several illnesses that are chronic and acute are becoming more relevant as the world’s aging population expands, and the medical sector is transforming rapidly, as a consequence of which the need for “point-of-care” (POC), identification/detection, and real time management of health issues that have been required for a long time are increasing. Biomarkers are biological markers that help to detect status of health or disease. Biosensors’ applications are for screening for early detection, chronic disease treatment, health management, and well-being surveillance. Smart devices that allow continual monitoring of vital biomarkers for physiological health monitoring, medical diagnosis, and assessment are becoming increasingly widespread in a variety of applications, ranging from biomedical to healthcare systems of surveillance and monitoring. The term “smart” is used due to the ability of these devices to extract data with intelligence and in real time. Wearable, implantable, ingestible, and portable devices can all be considered smart devices; this is due to their ability of smart interpretation of data, through their smart sensors or biosensors and indicators. Wearable and portable devices have progressed more and more in the shape of various accessories, integrated clothes, and body attachments and inserts. Moreover, implantable and ingestible devices allow for the medical diagnosis and treatment of patients using tiny sensors and biomedical gadgets or devices have become available, thus increasing the quality and efficacy of medical treatments by a significant margin. This article summarizes the state of the art in portable, wearable, ingestible, and implantable devices for health status monitoring and disease management and their possible applications. It also identifies some new technologies that have the potential to contribute to the development of personalized care. Further, these devices are non-invasive in nature, providing information with accuracy and in given time, thus making these devices important for the future use of humanity.

## 1. Introduction

In recent years, medical industry has changed quite a lot to from mere conventional devices to devices that are smart in nature. These devices are generally based on biosensors (bioanalytical devices that have the recognition element and a transducing system) or other sensors (such as piezoelectric, optical, micro-electro-mechanical systems, field effect transistor, ultrasonic based, capacitive sensor, etc.) and can be used for health monitoring, detection, and prevention of many diseases or disorders [1,2,3]. They provide point-of-care and personalized monitoring. Due to the global market shifts to more of such devices, the Compound Annual Growth Rate (CAGR) of such devices is expected to grow to 38 percent of the global market share from 2017 to 2025 [1].

Smart devices such as wearable biosensors are non-invasive in nature and provide real-time monitoring for the patient at any given point of time [4,5,6]. Such technologies can be a powerful tool in disease management and change of behavior of health. Studies have been conducted showing positive results in disease management where viral load and related risks associated to it were reduced. Furthermore, in lung transplant patients, a PDA (personal digital assistant)-based assistance improved perceived self-care agency ratings [7]. Wearable biosensors are biological sensors that are attached to the body of a person in a comfortable manner, such as clothing, bandages, watches, glasses, contact lenses, and rings, and provide features that set them apart from conventional devices in the perspective of mobility or portability, simplicity of use, and adaptation to the environment [8]. Accessories, integrated clothes, body attachments, and insertions have all been created as wearable devices over time. Ingestible devices are also promising technology for improving health outcomes that may, for example, be useful in monitoring, diagnosing, or releasing drugs at specific sites inside the body. Portable devices can be in the form of small handheld devices that can operate at any location. They may even be temporarily attached to the human body as peripheral devices/accessories. To a certain extent, portable systems can be similar to wearable biosensors in terms of their monitoring capability, the functionality they provide, and the services they can be combined with. The evolution of devices that are implantable, which makes diagnosis as well as prognosis possible using biomedical devices as well as small sensors, has considerably improved the effectiveness and quality of health care over the last several decades, thanks to remarkable advances in electrical, biocompatible materials, and nanomaterials. In recent years, cardiac pacemakers have been developed such that the early pacemakers which were external are now internal with controllers [9]. Due to the emergence of smart devices and the emerging mobile devices for medical use, health care services have also evolved; such services are known as mHealth (mobile health) services. According to the World Health Organization, 114 member states (83 percent) reported providing at least one form of mobile health service. Many nations, on the other hand, provided four to six options. Health call centers (59 percent), emergency toll-free telecommunications facilities (55 percent), emergency and disaster response (54 percent), and mobile telemedicine (49 percent) were the four most commonly recorded mHealth programs [10].

**Figure 1:**
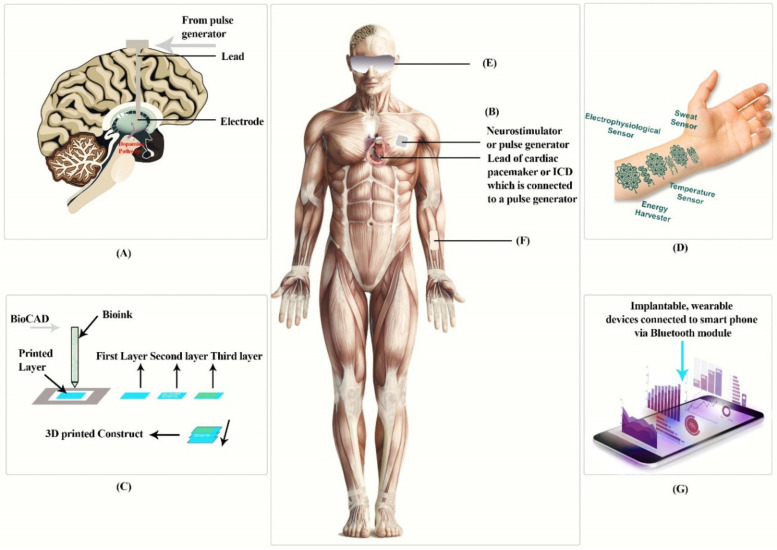
The above figure represents implantable devices: (**A**) Deep brain simulation, (**B**) implantable cardioverter defibrillators, (**C**) Boink, and (**D**) tattoo, and wearable devices: (**E**) contact lens, (**F**) dermal patch, (**G**) devices connected to a smartphone. This diagram is redrawn from [1,18,19,20].

In this paper, we discuss smart devices [11,12] which can be classified as wearable devices [13], portable devices, implantable devices [11], or ingestible pills [14,15]. Wearable devices are non-invasive, real-time biosensors that allow for continuous monitoring of humans, providing enough data to determine health conditions and even provide a preliminary medical diagnosis [1]. Portable devices are devices that have miniaturized biosensors which are portable in nature, can be used for health monitoring, detecting infectious and non-infectious diseases, and providing early diagnosis and POC (point of care) [16,17]. Implantable devices are those biocompatible devices that can be implanted inside the body, having a controller to monitor or regulate body functions, such as implantable cardioverter defibrillators (Figure 1) [9].

**Figure 2:**
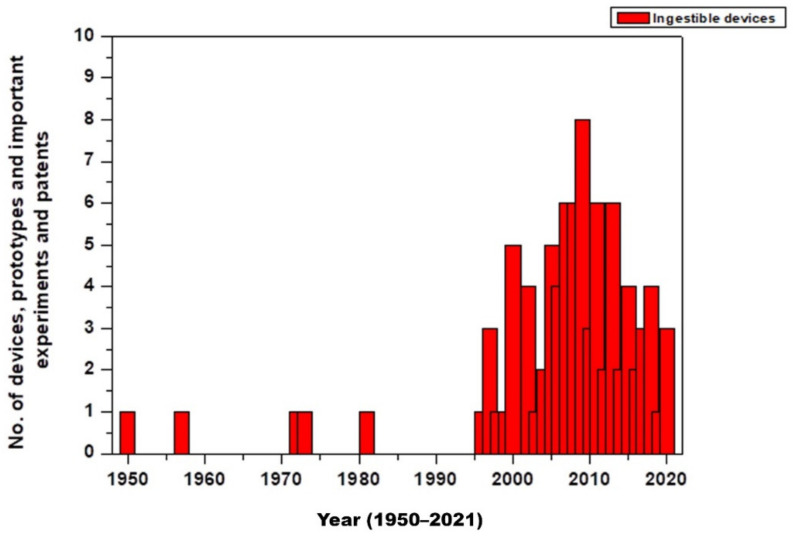
No. of devices, prototypes, important experiments, and patents [14,21,22,23,24,25,26,27,28,29,30,31,32].

Ingestible pills are safe and a non-invasive approach to monitoring, diagnosing, or releasing drugs at a specific site inside the body. They are used to monitor pH, temperature, blood, other body enzymes etc. These pills are biocompatible in nature. The initial development of such pills was in the 1950s. Since the approvals from the regulatory bodies in the US and Europe in 2000, there has been a rise in the development of such ingestible biosensors. Below is a graph representing the development of such pills (Figure 2) [21].

## 2. Wearable or Attachable Devices

The next era of personal as well as portable health care technology for remote medical progress is attachable or wearable monitoring gadgets or devices. Adaptability and flexibility similar to that of the skin, which provides sensing that is accurate and dependable without affecting a user’s natural mobility and ease, is a key attribute that defines an attachable device [33]. Guk et al. [1] classified wearable skin patches and contact lens as wearable devices (Figure 1), which we examine further in this review.

### 2.1. Wearable Skin Patches

Body patches that can be worn are becoming more popular in the wearables industry. Electronic sensors that are soft, versatile, and stretchable are attached to the soft tissue to provide a new medium for robotic input, monitoring, and continuous health care can be called wearable or attachable skin patches [34]. Since skin patches may be hidden by clothes and capture more precise data without being interrupted by movement, they are perfect wearables. Temperature, strain, sweat, and cardiovascular monitors have been utilized for human skin wearing patches.

It is really critical to monitor the cardiovascular signaling of patients seeking medical treatment via blood, heart rate, and blood pressure monitoring. A continuous blood pressure monitoring device sensor that is thin, flexible, and patch-like has been developed, along with a specially designed system for ferro-electric film, special electrodes, and electrical circuitry that are flexible, to ensure measurements on the human chest for electrocardiogram (ECG) and ballistocardiogram (BCG) at the same time [35].

A portable patch sensor with (adjustable) flexible piezo-resistive sensor, also called FPS, and epidermal-ECG sensors have been developed to measure blood pressure (BP) without a cuff [36]. The system monitors the ECG and epidermal-pulse signals simultaneously, with the pulse transit time (PTT) approach providing moment-to-moment BP data instantaneously. The FPS detection mechanism was constructed using a parametric model, and the operating parameters were optimized, resulting in a highly steady surface pulse signal. This sensing patch is particularly suitable for ultra-low power (3 nanowatts) and senses subliminal physiological changes including after or before exercises to offer suitable solutions that are promising to track BP in real-time and at home [1].

#### 2.1.1. Monitoring of Body Fluids

Sweat is particularly significant as a typical bodily fluid because it includes a huge number of key indicators, such as electrolytes, tiny molecules, and proteins. Wearable sensors for sweat analysis have been developed in recent years and have identified numerous sweat components [37]. Such sensors may have an inductive coil and planar capacitors and certain soft substrates that can absorb body sweat through capillary forces, thus making them able to analyze the components of the sweat such as OH^−^, H^+^, Cu^2+^, Fe^2+^ through colorimetric detection methods [1]. A potentiometric sodium ion sensor was developed to deposit a polyvinyl chloride membrane in the internal layer of electrochemically deposited Poly,3,4ethylenedioxythiophene (PEDOT) and the pH sensing layer dependent on high iridium oxide (IrOx) membrane sensitivity. The doping enzymes at the end of a copolymer membrane with outer layers of outer polyurethane have strong selectivity when various analytes are present in the amperometric-based lactate sensor. [38]. Furthermore, there is another device called the PDMS dermal patch, a dermal patch made of polydimethylsiloxane (PDMS), which is a thermal ablation microfluidic sampling device technology that provides non-invasive control as well as sampling of biomolecules such as glucose or others that are found in interstitial fluids without the need for invasive extraction. Thus, it is a great help in monitoring glucose for a diabetic patient [39].

#### 2.1.2. Monitoring Body Temperature

During the early stages of diagnosis and treatment of a condition, it is critical to monitor variations in skin temperature [40,41]. Researchers have created a patch that monitors human stress in a flexible way and has a smaller area of contact with the skin, making it possible to increase patch wear and lessen the harmful impact created by psychological stress on human society as well as human health. This human stress monitoring patch is composed of three sensors which can monitor skin temperature and conductance, as well as pulse wave, in a stamp-sized format (25 mm × 15 mm × 72 mm). Skin contact areas were reduced by 1/125th of the standard single layer multiple sensors thanks to the development of multi-layered integrated structures and associated micro-manufacturing methods or processes. The new innovation of a lightweight pulse wave form sensor built of flexible piezoelectric membranes, aided by (perforated) polyimide membranes along with a high degree of chemical resistance and flexibility, has increased the patch’s flexibility. These assembled patches had 0.31 Ω/°C of sensitivity for the human physiological spectrum as well as a pulse response time of 70 ms for measuring skin temperature [42]. Moreover, TS (transparent and stretchable) sensors that detect small variations in skin temperature as well as deformation throughout human activities have been simplified into an easy procedure that can be conveniently applied to an individual or the body as a patch. Temperature-sensing devices that were TS-gated and TS-resistive had a high sensitivity of around 1.34 percent/°C, and after one thousand stretching cycles with a 30 percent strain, there was no difference in response [43].

### 2.2. Contact Lens

Contact lenses that are smart can non-invasively detect the physiological details of both the eyes as well as tears from the eyes. Several types of contact lenses have been created to track the chemistry (glucose and lactate) and tear fluid’s electrical conductivity, as well as transcutaneous gases present in the eye’s mucous membrane, using optical and electrical techniques. Alexeev et al. [44] developed photonic crystals consisting of colloidal particles submerged in hydrogel in a face-centered cubic configuration for non-invasive glucose monitoring of tear fluid. Photonic glucose-sensing material sensed glucose in the range of the 100 μmol/L concentrations found in tear fluid. The detection limits were ~1 μmol/L in synthetic tear fluid.

A fluorescent contact lens was proposed by March et al. [45] along with a hand-held photo-fluorometer. Tetramethyl rhodamine isothiocyanate concanavalin A, also known as TRITC-Con A, and fluorescein isothiocyanate dextran, also known as as FITC-dextran, combined in liquid hydrogel nanospheres were used to make the contact lenses. If the glucose concentration rises, the FITC-dextran on TRITC-Con A is displaced from its combined position, raising the fluorescent strength and intensity.

The electronic enzyme L-lactate sensor which was developed by Thomas et al. [46] detects L-lactate present in tear fluid using contact lenses and is designed to be as non-invasive as possible. Its sensor features a functional platinum structure with glutaraldehyde, lactate oxidase, and bovine serum albumin cross-linking, and it is coated in medical polyurethane. Based on an immediate time to respond of around 35 s, an average sensitivity of ~53 µAmM^−1^ cm^−2^, which is within a linear range, and required resolution, the sensor on the tear film is able to accurately determine the physiological lactate concentration (i.e., L lactate concentration).

### 2.3. Other Wearable Devices

Another wearable device, introduced recently, is the mouthguard to analyze salivary uric acid using integrated wireless electronics [47]. In another study, the mouthguard was able to detect and monitor glucose from saliva [48]. Another device, a trilayer radio frequency tooth-mounted sensor which is used to monitor food consumption, is made possible when it is functionalized with analyte sensitive layers. The dielectric sensor is made up of biocompatible materials that may be put in the mouth, which can also detect alcohol, pH, salinity, sugar, and even temperature [49,50]. Other wearable devices are used for musculoskeletal applications as well as for thermotherapy [51].

## 3. Implantable Devices

The number of people who have been treated with implanted electronic devices for cardiovascular disease, such as implantable cardioverter defibrillators (ICDs), as well as implants such as deep brain stimulators (known as DBS) for Parkinson’s Disease, etc., has been growing since the 1960s, when cardiac pacemakers were initially introduced [52]. The majority of implanted devices are made up of batteries, biocompatible materials, and programmable circuitry. There are several implantable devices (Figure 1A–D), of which the most popular are implantable cardioverter defibrillators, bioinks and 3D print implants, and deep brain stimulators.

### 3.1. Implantable Cardioverter Defibrillators

The majority of implanted devices are made up of batteries, biocompatible materials, and programmable circuitry. A pacemaker device is used in the treatment of arrhythmias, or abnormal heartbeats, by delivering electrical pulses with a low energy. Thus, when abnormal heartbeats are recognized, they restore normal rhythm. The ICDs (implantable cardioverter defibrillators) are the most recent form of the pacemaker, and they work in a similar way. Half of all fatalities arising from heart disease are caused by sudden cardiac death (SCD). An ICD has the ability to provide a high-energy electric pulse if a traditional pacemaker is unable to restore the usual heart rate rhythm. Indeed, in patients with a high risk of SCD due to ventricular arrhythmias, the ICD is linked to substantial decreases in death [1].

### 3.2. Bioinks and 3D Print Implants

Biosensitive inks have been created by Harvard and MIT researchers known as bioinks (similar figure as shown in Figure 1) [20]; they use a basic chemical reaction to process and transmit information and data without using energy, showing the use of the body’s surface as an interactive display or a biointerface [53]. Traditional tattoo inks have been phased out in favor of color changing biosensors because the fluid of the interstitial fluids, also known as the tissue fluid, varies. Since the substance blends very closely with the plasma, it is a reliable indicator for the chemical composition in blood anywhere at a given point in time. The researchers looked at four biosensors that use color-changing inks to measure glucose, pH, and sodium levels on the skin. To date, pig skin has been used to investigate two kinds of inks in vitro. One is used to track glucose levels present in the patient’s blood, changing from green to brown as the level rises. When an increase in sodium can be seen, the second type turns bright green and brings down sodium levels in order to avoid dehydration. Another study was reported in which a 3D bio-printing hydrogel ink was made; the 3D bio-printing hydrogel ink was capable of printing a programmed bacterial cell with a resolution of around 30 micrometers on a massive 3 cm-sized biomaterial [1]. In this 3D printing ink, a mixture of polymeric micelles and photo initiators are mixed with waterborne, nutrition, programmed bacterial cells and signal chemicals. Initially, a multi-hydrogel ink made up of many different types of cells or chemicals is used, followed by UV irradiation of the printed substance, to conduct direct 3D printing on a biomaterial. The engineered bacteria cells can see the new features of the device through 3D printing. Live tattoos are printed on an elastomeric sheet consisting of a double layer of cells (responsive to AHL-N-Acyl homoserine lactone, IPTG- isopropyl beta-D-1-thiogalactopyranoside, or Rham-Rhamnose). When the chemical is administered, the tattoos emit green fluorescence in the matching 3D print pattern [54].

### 3.3. Deep Brain Stimulation

Deep brain stimulation or DBS is a neurosurgical technique which allows for circuit-based neuromodulation in specific areas of the brain. DBS is the most standard of treatments for Parkinson’s disease, dystonia, and essential tremor. It is also still being studied for other disorders including abnormal circuitry, such as Alzheimer’s disease as well as major depressive disorders. DBS devices of the present day, which are based on cardiac devices, have an intracranial electrode, an extension wire, and a pulse generator, and now have been developed steadily over the last two decades. Tech and imaging advancements, as well as a better understanding of brain conditions, are poised to change how DBS is perceived and administered to patients [55].

### 3.4. Other Implantable Devices

Vega et al. developed nanosensors made of graphene that do not require a power source and can identify and track microorganisms such as bacteria in the saliva or breathing using biomaterials including tooth enamel [53]. Pathogenic bacteria can be detected at a single cell level using graphene nanosensors that use antimicrobial peptides. Jia et al. [56] recently created a non-invasive system that is useful for monitoring the occurrence of glucose, lactate, alcohol, and ammonia in the body based on a tattoo. Tattoo biosensors, that can quantify the levels of concentration of lactate in a non-invasive manner in the human sweat, were designed and created to detect or monitor electrochemical signals that the enzymes produce. The novel lactic acid oxidase-functionalized skin biosensor demonstrates a high linear specificity of up to 20 mM for lactate that is secreted by the sweat glands. Furthermore, since the tattoo sensor is versatile, it has a long lifespan even though the skin moves often. Sensors have been used to analyze the difference in lactate levels in the sweat glands of participants involved in long-term repetitive exercise in real time. An ammonia potentiometric tattoo tracker, on the other hand, employs a non-actin ionophores-based ammonium specific polymer membrane and a solid-state reference electrode. Using a tattoo biosensor with an ammonium selective polymer membrane, it was evaluated in physiological testing that NH4+ levels were of 0.1 to 1 mM [57]. Another device which works on enzymes that has been developed is a tattoo-based blood sugar detecting device, which is made up of a reverse osmotic pressure-derived epileptic glucose as well as an enzyme-based current tracking biosensor; it uses an oxidized Prussian blue converter. The specific response of this sensor is up to 3 µM and it is sensitive to concentrations of glucose of around 23 nA/M. Placing the sensor on the subject’s skin or monitoring variations in glucose levels of the blood as they ate were both part of the validation procedure. This study’s results suggests that the tattooing systems which combine biosensing and iontophoresis might be beneficial in the treatment of diabetes [58].

## 4. Ingestible Pills

**Figure 3:**
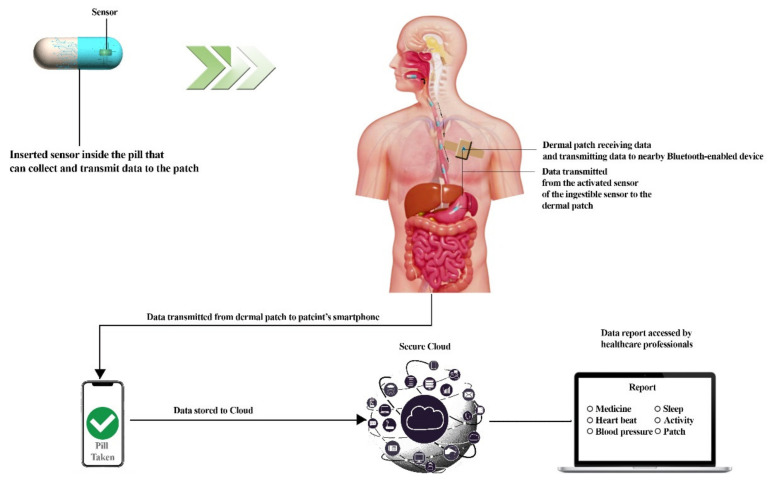
Working diagram of an ingestible pill which represents the components of ingestible pills, the dermal patch which helps in receiving and transmitting data, data stored in the Cloud, and final assessment by a health official. This diagram was redrawn from [61,62].

The ingestible sensor or pill may pass through the digestive tract’s lumen and reach organs in the belly. As a consequence, in addition to intrinsic genital contents and luminal fluid, the ingestion sensor gathers and delivers biometric data on enzymes, hormones, electrolytes, microbial communities, and metabolites in the proximity of the organs. Thus, using an ingestible sensor to obtain the fluid that one may wish to detect is a safe and non-invasive option (Figure 3) [59]. In the last decade, there has been significant progress of such pills. Thus, they are useful for many purposes. One example is a gut microbiome redox sensor that monitors the gut by monitoring oxidation redox potential to evaluate the oxidation state of the gut; the device has been tested in a rat but in vivo results of the device are awaited as it undergoes such trials [60]. Below, Figure 3 represents a functional diagram of most ingestible pills present today.

Further, based on the functions of such pills, they may be categorized as imaging capsule, temperature-sensing capsule, pH monitoring and pressure-sensing capsule, multifunctional advanced capsule, gas sensing capsule, ultrasound imaging, or electro-chemical-sensing [14].

### 4.1. Imaging Capsules

The focus was originally on endoscopy, which still accounts for the majority of the ingestible device industry. The motivation behind this was to discover a way to somewhat replace the tube endoscopes, which are used in invasive endoscopies, put into the mouth or rectal orifices with a less invasive approach [63]. PillCam ESO PillCam COLON and PillCAm SB2 developed by Imaging Inc are some examples of it. PillCam passes through the esophagus, has a high frame rate, and contains two cameras at each end. Pill Colon is used for wide angle imaging and for colonoscopies (complementarily) [64].

### 4.2. Temperature-Sensing Capsule

Body temperature is routinely measured with ingestible temperature sensors, mostly to assess patient’s heat stress, perhaps in an industrial worker or a military soldier [65]. Core temperature pill is a thermistor-based detection of temperature developed by Vital Sense [66].

**Figure 4:**
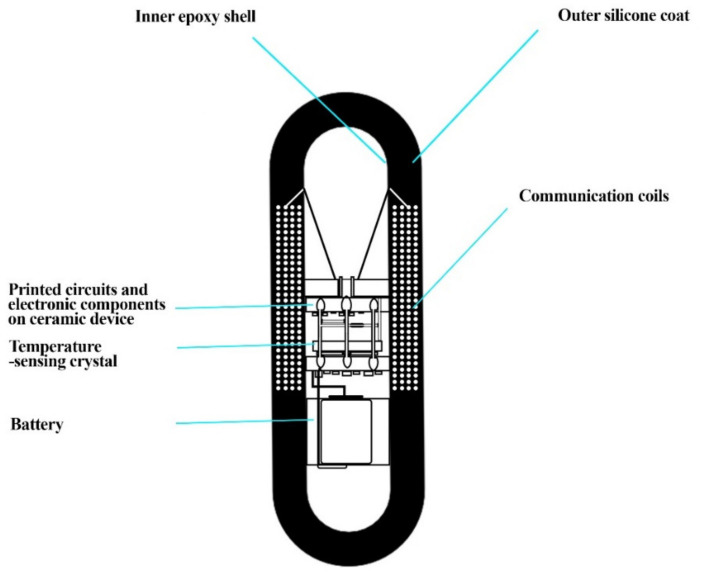
The ingestible thermal monitoring system showing inner epoxy shell, outer silicone coat, printed circuits, temperature-sensing crystal, and communication coils. This diagram was redrawn from [67].

A pill was developed by NASA (National Aeronautics and Space Administration) in collaboration with John Hopkins University in the late 1980s. This pill was named an ingestible thermal monitoring system (Figure 4) and was consumed by US senator and astronaut John Glenn to monitor temperature, since space has extreme temperatures from 250 °F to −250 °F. The suit also releases heat which may cause heat stress. As the pill is swallowed, the quartz electrode vibrates at a frequency that corresponds to the body’s temperature, sending a low-frequency pulse through the body that is completely harmless. This signal can be read by an external recorder, which can show a core body temperature and other vital statistics. The pill travels through the digestive system comfortably after 18 to 30 h. Thus, the idea of an ingestible pill was promising for the scientists and researchers at NASA [67].

### 4.3. pH Monitoring and Pressure-Sensing Capsule

Due to the production of HCl (hydrochloric acid), the gastric fluid inside of the stomach is very acidic. The transit of the ingestible capsule to the alkaline duodenum is marked by a sudden pH increase (>3 pH units) from the gastric baseline. The ileocecal junction (where the ileum meets the colon) is usually recognized by a pH drop of at least 1 pH unit. During passive progression, such capsules often monitor pH at the junction [68]. The capsules usually last many days, after which the test is then carried out while the patients continue to follow their normal meals and activities [69]. These pills can measure the pressure of the GI (gastrointestinal) tract too; however, much improvement is required [70]. The Bravo pH system, by Imaging Inc., can track the GI in passive progression and helps monitor GERD (gastroesophageal reflux disease), small bowel dysfunctions, functional non-ulcer dyspepsia, colonic disorders, and pressure [67].

### 4.4. Multifunctional Advanced Capsule

The capsule is equipped with multiple functions; thus, multiple sensors are a part of it. Several sensors and systems, such as pH sensors, temperature sensors, fluid pump, drug reservoir, etc., are present in such capsules [71]. Developed by Philips Electronics Inc., Amsterdam, The Netherland, it contains a pH and temperature sensor, radio frequency wireless transceiver that receives commands, a fluid pump, and a drug reservoir. It monitors and release drugs on command [72].

### 4.5. Gas-Sensing Capsules

Gas sensor capsules are a relatively new addition to the market for ingestible electronic capsules. Sensing gases produced by gastrointestinal processes as by-products is a unique concept for monitoring gut functioning [73,74]. The gas sensors function in both anaerobic and aerobic environments and are usually protected by the gas permeable membranes with excellent integrity. Some gases are generated in the stomach as a consequence of enzymatic and natural chemical reactions. Chemical interactions lead to substantial alterations in the gas profiles of O_2_ and CO_2_ in the stomach. The vast bulk of gas production in the small intestine (jejunum and ileum) and colon is caused by bacteria. These bacteria create SCFAs (short chain fatty acids) by fermenting undigested and unabsorbed dietary substrates, also in tiny amounts. There are also odorous by-products (sulfide-containing gases such as H_2_S or hydrogen sulfide), as well as hydrogen, carbon dioxide, and methane. Since the increase in amount of such gases also means an increase in the bacterial population, by monitoring these gases, the capsule helps us to understand the bacterial population in the gut, thereby improving health monitoring and treatment [73].

### 4.6. Ultrasound Imaging

Generally, such capsules such as Sonopill comprise ultrasound transducer arrays and an ultrasonic receiver/transmitter circuits-specific IC (integrated circuit). They are fitted with four piezoelectric transducers with discrete units with a 15–50 MHz operating frequency in the capsule. They are useful for ultrasound imaging [75]. Memon et al. [30,31] developed one such pill and further developed it in the next year with CMUT array with PDMS-filled trenches with a wireless transmitter. The device is meant for capturing ultrasound images of multiple layers of the GI tract. Wang et al. [76] developed another such pill with three components: a cylindrical capacitive micromachined ultrasonic transducer (CMUT), imaging circuitry, and a wireless transmitter; the pill was useful in monitoring multiple layers of the entire GI tract.

Other techniques involve contrast-enhanced ultrasound, which depends on encapsulated microbubbles filled with gas. It has a unique signature in an acoustic field. Such technique is used for different medical imaging, useful to study the morphology and functions of internal organs. Indeed, molecular events too can be studied using this technique [76]. Recently, there was a study made on hybrid shell droplets encapsulating decafluoropentane (DEF). The deposition of a dextran methacrylate layer onto the surface of surfactants is used to make the droplets. UV curing, which introduces cross-links in the polymer layer and transforms the shell into an elastomeric membrane with a thickness of around 200 nm and viscoelastic behavior, has stabilized the droplets against coalescence. The droplet can be used as ultrasound contrast agent, helpful in diagnosis. Importantly, irradiation with ultrasound causes the DFP of the droplets to evaporate, turning the particles into microbubbles. The existence of a durable cross-linked polymer shell gives the droplets extraordinary resilience, even during the core phase transition, allowing them to return to their original condition after switching off the ultrasound [77]. Further improvements have also been observed in recent times using this technique [78]. Apart from this, one of the most important techniques based on ultrasound is photoacoustic imaging. It is a non-invasive bioimaging technique using ultrasonic waves by irradiating a material with a pulsed LASER, and it reconstructs a picture of the tissue’s light energy absorption pattern. Above all, this unique imaging modality provides useful optical contrast that displays anatomical, molecular, functional, and even histological information [79].

### 4.7. Electro-Chemical Sensing

It is made up of a multielectrode sensor with voltammetry-capable potentiostatic circuits [59]. McCaffrey et al. [28] developed a device which consists of a switch, batteries, wireless module, micro-controller, and electronic circuits. It is based on chemicals in the lumen liquid and such devices generally follow an algorithm based on the Nernst Equation.

## 5. Portable Devices

They are devices that are portable in nature. For our study, we further looked at a number of reports and came to the conclusion that portable devices can be used for both infectious [16] and non-infectious diseases [17], as well as to monitor health. Further, we describe various portable devices, their functions, and their advantages.

### 5.1. Portable Devices for Health Monitoring

**Figure 5:**
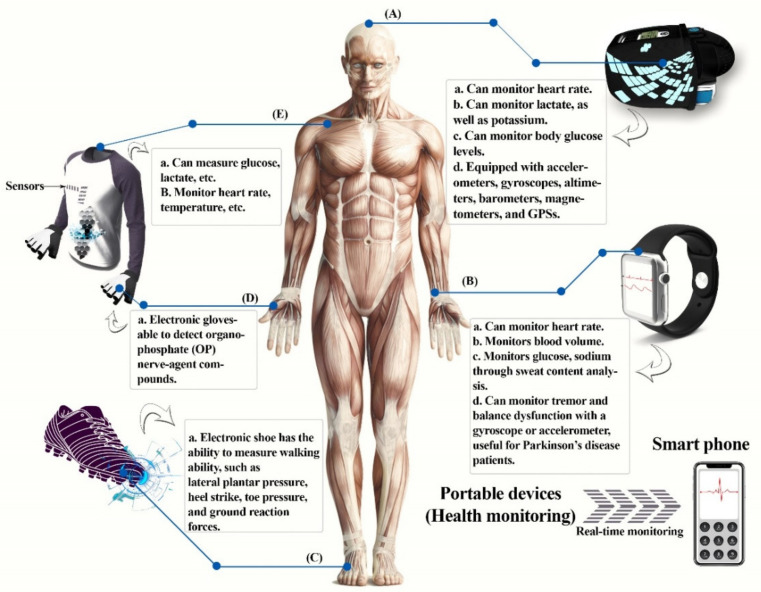
Portable devices for health monitoring. (**A**) Head-mounted devices, (**B**) wrist-mounted devices, (**C**) electronic shoe, (**D**) electronic gloves, (**E**) e-textiles or smart clothing. This diagram was redrawn from [1].

Portable devices can be used for monitoring blood pressure, volume, glucose, and heart rate. They can also be used in combination with smartphones, laptops, etc. Various types of such devices can be categorized under head-mounted devices, wrist mounted devices, e-textiles, etc. (Figure 5A–E) [1], which we study further.

#### 5.1.1. Wrist-Mounted Devices

Commercially available wrist-mounted physiological tracking systems have improved battery life and hardware miniaturization for translating raw signals to interpretable data in real time, for example, smart watches and fitness bands, transitioning from simple accelerometer-based “smart pedometers” and toward biometric sensors. Non-invasive tracking devices normally perform two tasks: (1) monitoring human physiological and activity signals, and (2) communication with electronic devices [3,80].

S. S. Lee et al. [81] created a portable system with a Hall device that can monitor minimal variations in the permanent magnet’s magnetic field and collect pulse-wave data. This is a pulsimeter without a cuff and can be worn on the wrist. Hsu and Young [82] demonstrated a personal wearable health monitoring device with skin-surface coupling that tracks elevated BP (blood pressure) wave patterns instantaneously and connected to portable gadgets such as smartphones and laptops.

Ishikawa et al. [83] developed a bracelet-style PPG (photoplethysmography) heart-rate sensor that detects or monitors variations in the heart rate of a person and shows the probability of resolving artefacts of motion in everyday activities [3].

One of the most prevalent forms of portable wearable devices is smart watches. GlucoWatch^®^ biographer was the first commercial smart watch to be approved by the FDA (Food and Drug Administration) which has a glucose sensor available for sensing glucose non-invasively. It electrochemically acquires information about glucose concentration extracted by reverse iontophoresis from skin interstitial fluid [84].

Smart watches with a gyroscope or accelerometer can be used to analyze balance as well as tremor dysfunctions present in patients with Parkinson’s disease (PD). Lopez-Blanco et al. [85] looked at smart watches for tremor quantification in Parkinson’s patients and acceptance clinical correlation and usability as a tool for monitoring. Consequently, the usage of smart watches as a clinical tool is a possibility and it has a high level of patient approval. Tison et al. used smart devices to develop an algorithm to detect atrial fibrillation (AF) from the heart rate data measured with PPG sensor and step count with the accelerometer [86]. The main cause of stroke is AF, and patients at risk of stroke can prepare for the disease by continuously monitoring AF.

#### 5.1.2. Head-Mounted Devices

Smart glasses with a built-in display are a form of head-mounted display device that shows data or information [1]. The smart glasses created by Constant et al. [87] are eyeglasses featuring a photoplethysmography (PPG) sensor on the nose pad that continuously measures heart rate; they are basically pulse-sensing eyeglasses. Sempionatto et al. [88] demonstrated eyeglasses with a nose pad that have a lactate-biosensor for lactate monitoring and an ion-selective electrode made of potassium for measuring potassium ions in sweat instantaneously. Arakawa et al. [89] using microelectromechanical systems (MEMS), as well as using Ag/AgCl and Pt electrodes produced with enzyme-membrane immobilized glucose-oxidase. They developed a “mouthguard glucose sensor”. Another enzyme-based biosensor was developed to measure salivary uric and lactate. Kim et al. employed wearable salivary metabolite biosensor based on the integration of a printable enzymatic electrode on a mouthguard. Using complete human saliva samples, the new mouthguard enzymatic biosensor, which is based on an immobilized lactate oxidase and a low potential detection of the peroxide product, exhibits high sensitivity, selectivity, and stability [90].

#### 5.1.3. E-Textiles or Smart Clothes

Smart textiles, also known as smart clothing, are made up of conductive devices that are attached to or woven into clothing [91,92]. Three elements are needed in smart textiles: a sensor, an actuator, and a monitoring device. For example, physical activity and human-physiological signals, biomechanics, body acceleration such as motion, and pressure, are monitored using e-textiles, which include electrodes [80,93].

Liu and Lillehoj [92] devised a system for detecting glucose or lactate; the system is built into a fabric utilizing lactate-oxidase- and glucose-oxidase-based electrodes to accurately assess lactate or glucose. To offer materials with desirable functions, using hydrogel-elastomer hybrids and genetically modified bacteria as well as genetic circuits, Liu’s team created living material and a glove.

During daily exercise, the wearable vest “HexOskin” can track heart rate as well as breathing rate [94]. The ability to walk has been quantified using an electronic shoe, which monitors ground reaction forces, toe pressure, heel strike, and lateral plantar pressure, which are important for determining gait phases [95,96]. In order to identify organophosphate (OP) nerve-agent compounds, Mishra et al. [97] created a glove that comprises an electrochemical biosensor and a printable stretchable electrode which is enzyme-based. On the index finger of the glove, there is a carbon-based counter electrode, active electrode, a thumb-printed carbon pad, and a reference electrode based on Ag/AgCl. Here, the thumb is considered a collector/sampling finger, while the index finger has a layer of organophosphorus hydrolase and functions as a detecting finger. Moreover, stress-resistant inks have been utilized for the electrode system to print as well as for the lengthy serpentine connections to the wireless electrical interface. The “Lab-on-a-glove” is used as a POC screening device and also for defense and security of food applications.

#### 5.1.4. Other Portable Devices for Health Monitoring

In the recent years automated insulin pumps have been introduced; the portable device is a great help for the diabetic patients. [98] In recent years, digital stethoscopes have also been introduced with the ability to convert acoustic signal to digital [99]. Further in the field of otoscopy, hand-held otoscopes are being developed, helping patients and physicians with an easy and quick remedy for those patients having problems in the ear [100].

### 5.2. Portable Device for Detecting Non-Infectious Diseases

Non-infectious diseases are those that are not caused due to pathogens and are not transferable to other individuals or are non-communicable diseases [101]. Portable devices are useful for detecting non-infectious diseases such as cancer. These include detection through analytical devices such as biosensors [102] as well as other devices such as electronic nose or gas chromatography time of flight mass spectrometry [17], which we discuss in detail.

#### 5.2.1. Biosensors and Ovarian Cancer

Cancer is a highly complex and complicated disease which is the result of a multi-step carcinogenesis process involving multiple physiological systems of cells, such as cell signaling and death. Cancer begins at a stage of a localized disease, but it is vulnerable to spreading to other parts of the body, rendering it incurable [103]. Ovarian cancer is one of the most common causes of death among women worldwide. This is one of the deadliest gynecologic cancers, owing to a lack of early detection strategies and the late onset of symptoms [104]. Ovarian cancer can be detected by the use of a portable device based on biosensors, Further, the team analyzed and divided biosensors on the basis of transducing element and biorecognition element, where for the transducing element it was divided further into electrochemical-, optical-, and mass-based biosensors. The biorecognition element is further divided into antibody, cell, phage, DNA (deoxyribonucleic acid), enzyme, and aptamers [102]. Further, the electrochemical biosensor is categorized as amperometric, potentiometric, impedimetric, or conductometric. Amperometric biosensor-based devices work when the biorecognition element is put on the surface of the device, which provides a substantial interface [105]. Aptamers are those that typically bind to a broad variety of cellular and molecular targets with a high sensitivity and specificity. They are affinity probes based on nucleic acids (RNA or DNA) that have several advantages over antibodies, including cheap cost, reusability, and temperature stability. Potentiometric biosensor-based devices are those that work with the help of electrodes, are ion-selective, and that produce an electrical response. Moreover, the authors found different immune responses that were helpful to detect ovarian cancer, such are tumor markers like HE4 (human epididymis 4) as well as markers such as CA125 (cancer antigen 125). Antibodies to detect different immune responses were helpful to detect cancer. In the devices with colorimetric biosensors, which, as the name suggests, may involve color changes, several nanoparticles such as gold nanoparticles and quantum dots are ideal and best to be used for such biosensors [106]. In optical biosensors, a device with a prism, light source, and thus optical detection limit was produced. The chip was made of silicon with gold film, such that the glass surface was below and the flow channel was above the metal surface. A1 apolipoprotein, TIMP3 (tissue inhibitor of metalloproteinase 3), and fibronectin were used as markers for detection [102]. Furthermore, mass-based biosensors include piezoelectric and magnetoelastic biosensors, such as acoustic wave biosensors. Piezoelectric quartzes or crystals are often used in mass-based biosensors for diagnostic purposes [107]. Quartzes are made to vibrate at a certain frequency using an electrical signal in this form of biosensor. A coating is applied to the outside or surface of the quartzes for the operation of sensing. The layer is made up of a biorecognition factor, such as an enzyme, antibody, or a cell, which has a precise binding approach with said analyte. Once the sample has been added, a precise binding occurs between the biorecognition elements on the device of the sensor and the analyte, resulting in a mass transition, which results in a change in frequency and the formation of an electronic signal [108].

#### 5.2.2. E-Nose and Colorectal Cancer

Colorectal cancer is one of the most lethal cancers found third most amongst men and second most in women globally [109]. Thus, detecting it in its early stages is prudent for any kind of further treatment. E-noses (electronic noses) have the ability to identify cancer early on. E-noses have benefits in comparison to technologies such as GC-MS (gas chromatography-mass spectrometry) since they can evaluate samples instantaneously, at cheap cost, and are portable equipment that are simple to use. The PEN3 is a portable olfactory device with dimensions of 92 mm × 190 mm × 255 mm used for chemical and gas detection. It is a sensor array and a gas sampling equipment combined. The PEN3 comes with an autosampler (known as HT2000H Dynamic Headspace Auto Sampler) that connects to the PEN3 software directly (WinMuster PEN version 1.6.2.18). The arrays of the sensor are made up of ten distinct thick film metal oxide sensors that operate at temperatures ranging from 250 to 550 degrees Celsius. The sample gas is pumped through the sensor array by one pump, while the other feeds the sensor array with filtered reference air or zero air. The system is also cleaned using zero air. As a baseline or reference gas, zero air is utilized, and the sensor response from the sample gas is compared to the reference gas. They used urine samples to detect cancer. A radar plot analysis taken from sensor responses were used in the study and it compared a non-cancerous cell line with the colorectal cancer cell line, which proved to be effective and possible to detect. Its specificity and sensitivity in separating cancerous from non-cancerous cells were further proven with neural network and random forest [17].

### 5.3. Portable Devices for Detecting Infectious Diseases

Infectious diseases are those diseases that can be easily transferred to other individuals. These diseases are caused due to pathogens and are fatal [110]. Thus, early detection is useful for treatment of such diseases. In order to facilitate early detection, various biosensors are used, which can detect such diseases quickly with a low limit of detection and high precision [111]. Further, the review includes such portable biosensors that are used to detect coronavirus, Zika virus, human immunodeficiency virus, human cytomegalovirus, and pathogenic agents causing malaria, tuberculosis, etc.

#### 5.3.1. COVID-19

**Figure 6:**
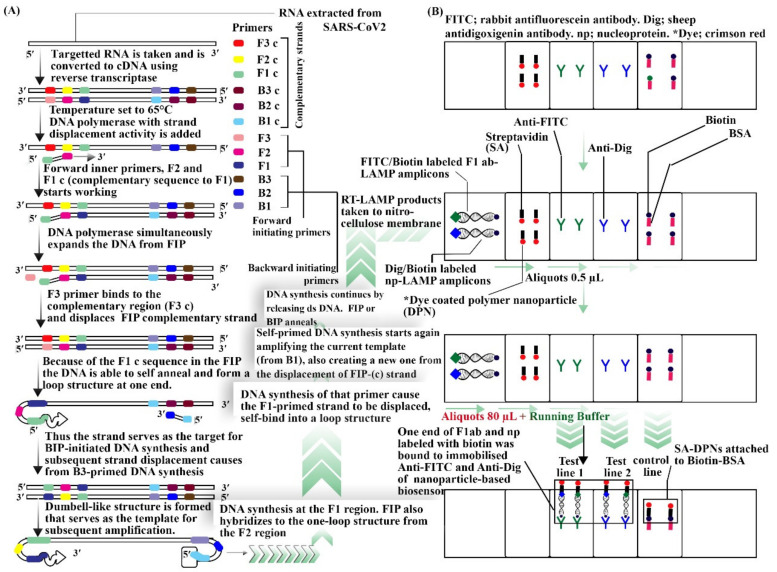
RT-LAMP NBS procedure and working diagram. (**A**) Procedure for reverse transcription loop mediated isothermal amplification. (**B**) Nanoparticles-based biosensor. This diagram was drawn from [111,116,117,118].

Coronavirus belongs to the Coronaviridae family, that is related to the sarbecovirus subgenus which is part of the Nidovirales order; it can be found in animals and may be transferred to humans [112]. Further, in a recent review on COVID-19, different conventional detecting methods for SARS-Cov2 were revealed, including reverse transcriptase polymerase chain reaction method, loop mediated isothermal amplification, lateral flow assay, and SHERLOCK (specific high sensitivity enzymatic reporter unlocking) based on CRISPR (clustered regularly interspaced short palindromic repeats) and serological tests. However, they expressed that these methods are not portable and a POC is needed. Thus, in the same paper, they reviewed different devices based on biosensors for detecting SARS-Cov2. These includes (a) field effect transistor (FET) [113], (b) nucleic acid-based biosensors [114], (c) optical biosensors [115], and (d) RT-LAMP-based biosensors. Biosensors for virus detection have been reportedly tested using specific transducers as a better alternative to the traditional assays. Biosensors are devices used for biological material analysis, which may directly include tissues, nucleic acids, cell receptors, enzymes, proteins, or derived samples such as engineered proteins, aptamers, recombinant antibodies, and so on, that are closely linked or grouped within a microsystem of physico-chemical transducers or transducers of various types such as piezoelectric, electrical, optical, and electrochemical. Under nucleic acid-based biosensor-based devices, genosensors such as compact tools were used. In addition, the use of LSPR (localized surface plasmon resonance) with nucleic acid was proven to be potent for further detection. For devices based on optical biosensors, the LSPR and hybridization technique was used which was detected with a spectrophotometer. RT LAMP NBS (reverse-transcription loop mediated isothermal amplification paired with nanoparticles-based biosensor) (Figure 6) is a device which was developed for the efficient detection of COVID-19 within an hour [111].

To determine whether SARS-CoV-2 genomic content exists, Alafeef et al. [119] designed a biosensor which is a device based on an electrical readout configuration for a graphene-based electrochemical biosensor. The biosensor’s selectivity comes from the incorporation of an appropriate architecture of thiol-modified antisense oligonucleotides (ssDNA) that is unique for the SARS-CoV-2 nucleocapsid phosphor protein (N-gene).

##### Zika Virus

**Figure 7:**
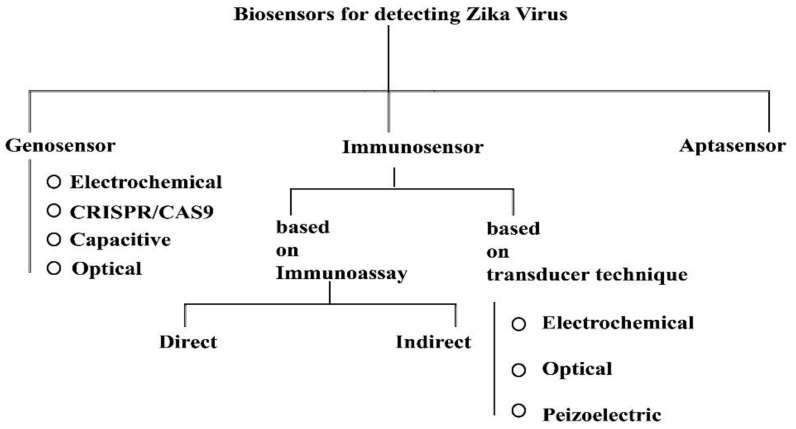
Types of biosensors for detecting Zika virus. This diagram was redrawn from [120].

Zika virus is caused through the bite of the Aedes species of mosquito; transmission may include blood transfusion, sexual contact, or from mother to fetus. Several conventional methods were reviewed recently that were used to detect the viral load or through mAbs, but recent studies stressed the importance of POC applications and devices based on biosensors that have played a huge role in detection of Zika virus. Further, there can be three types of biosensors useful for the for the detection of Zika virus; these include: (a) genosensor-, (b) immunosensor-, and (c) aptasensor-based devices (Figure 7) [120].

Devices with genosensors, those that are gene-based, use DNA immobilization techniques and different binding methods for, e.g., DNA-RNA, DNA-DNA, and DNA-protein to detect and analyze on the surface of the sensor [121]. Impedance and cyclic voltammetry are techniques that are common amongst electrochemical biosensors [122]. Cheng et al. [123] developed a device which uses a probe-based sequence specific capacitive sensing method with electrokinetics for the detection of Zika virus. Adegoke et al. [122] introduced a device that uses LSPR (localized surface plasmon resonance) with quantum dot nano-crystals on the plasmon surface, where four nanoparticles are activated by mercapto-propionic acids (MPA-Ag and MPA-Au), Au/Ag, or alloyed Au/Ag nanoparticles. Plasmonic NPs used were only for fluorescence in the Qdots. The hybridization technique was used for the desired RNA identification, and the identification was based on the fluorescence of the LSPR. Faria and Zucolotto [124] created a device that has a label-free electrochemical genosensor and it was used with disposable electrodes. Its morphological characterization was carried out using scanning electron microscope and atomic force microscopy. The probe DNA was immobilized or fixed in the electrode in this approach to detect Zika virus.

Devices with an immunosensor basically rely on the antigen–antibody interaction principle. Biological recognition can be any antigen or antibody; due to their specificity, they are called immunosensors since it involves antibodies. Here, the analyte combines with a particular antibody, thus one can recognize it very easily. It is vital to keep in mind that, here, the transducer is responsible for biophysical events being converted into electrical signal where the analyte binds to the antibody, thus it is digitalized by certain instruments [125]. Several techniques the such as electro-generated chemiluminescence (ECL) technique were used, wherein anti-Zika virus PSBs (polystyrene beads) generally bind or attach to the Zika virus in the sample. Another popular approach is to detect immunoglobulins IgG and/or IgM, where NS1 (non-structural protein 1) protein was immobilized and was used for biorecognition on the surface of gold nanorods (AuNRs), that act as a transducer. Whatman filter paper was utilized to immobilize NS1 protein by integrating it with AuNRs. As a protective element, a framework based on metal (ZIF-8) was employed [120,126]. Through a working interdigitated microelectrode of gold array, Kaushik et al. [127] showed the use of an electrochemical immunosensor to recognize the Zika virus protein. The mini-IDE (inter digitated electrode)-Au chip was prepared using specific ‘E’ (envelope) protein Abs (ZIKV-antibody) for Zika immobilization A. M. Faria and Mazon [128] designed a sensor where ZnO nanostructures immobilized on the printed circuit board (PCB) using ZIKV-NS1 antibody served as an electrochemical immunosensor. The immunosensor’s analytical responses were evaluated using cyclic voltammetry (CV). Takemura et al. [129] created a new immunofluorescence-based biosensor-based device for Zika virus NS1 detection. In a system that recognizes antibodies and antigens interaction, the sensor operates by amplifying the fluorescence intensity signal of QDs via LPSR signal to plasmonic gold nanoparticle.

Devices with aptasensors uses aptamers, which are tiny nucleic acids extracted via an iterative in vitro amplification and selection procedure from vast combinatorial oligonucleotide libraries. Systematic evolution of ligands by exponential enrichment, or SELEX, is the name of this selection technique [130]. A random oligonucleotide library (typically 1015–1016) is used to launch the process. Amplification, partition, binding, and elution are all part of the SELEX process.

#### 5.3.2. AIDS

HIV, or human immunodeficiency virus, causes AIDS, or acquired immunodeficiency syndrome. HIV predominantly affects CD4+ (cluster of differentiation 4) T cells, since those are its main target. HIV takes root in mucosal tissues after a transmission event, and it spreads to lymphoid organs within days [131]. The number of CD4+ T-lymphocytes per microliter of HIV-infected blood has consequences for prognosis and therapeutics, and it has been generally used to track disease progression as well as determine ART (anti-retroviral treatment). The host cells are CD4+ T-lymphocytes. By attaching or binding to the CD4 receptor via the HIV gp120 envelop glycoprotein, HIV infects and kills cells [132,133].

For POC diagnostics, Lee et al. [134] created a disposable (or non-reusable) RT-PCR chip, which was polymer-based, with pinched microvalves. This system was used to identify the HIV p24 (capsid protein) as well as gp120 main capsids and the envelop proteins that are encoded by the gag and envelop genes of the HIV for early HIV infection diagnosis. This solution, however, includes optimization for HIV-infected whole blood processing and standardized POC application in a resource-constrained setting for functional POC applications.

Further, Ozcan and Demirci [135] as well as SangJun Moon et al. [136] proposed and developed lens-less, portable, CCD (charge coupled device)-based microfluidic devices for HIV monitoring. Without the use of expensive machines, it counts CD4+ T lymphocytes in a microfluidic system which is made up of double-sided adhesive film, glass slides, and poly (methyl methacrylate) (PMMA). Without the use of any fluorescent marking or an optical microscope, the CCD sensor was used, and it was able to identify CD4+ T-lymphocytes, which are label-free and collected from a finger prick whole blood (10 µL) sample, using lens-less shadow imaging techniques. Then, it was counted by automated cell counting tools in a fraction of seconds [16].

#### 5.3.3. Detection of Human Cytomegalovirus

Human cytomegalovirus is classified under beta herpes viruses which affects human with lifelong infections. Recently, a review on HCMV discussed several POC devices based on biosensors such as electrochemical, DNA-based, optical, piezoelectric affinity, and immunosensors. Interestingly, the biosensor device based on piezoelectric sensor was based on strand displacement amplification technique [137], whereas the other biosensors were based on ellipsometry (device with 0.024 ng/mL of sensitivity) [138], sandwich ELISA (enzyme linked immunoassay), sandwich immunoassay, electrochemiluminescence, and other immunoassay principles fitted with immunosensors. A SPR (surface plasmon resonance)-dependent method for a label-free optical biosensor was also studied recently [139], whereas another team carried out a detailed study on DNA sensors that included EPAD (electrochemical paper based analytical device) integrated with zinc silver nano blooms [140]. Further, all these device’s sensors were effective, cost efficient, and provided quick detection methods which conventional devices fail to do [137].

#### 5.3.4. Tuberculosis and Other Disease-Causing Pathogens

Tuberculosis is an infectious disease that can spread via air (air-borne) and is caused due to *Mycobacterium tuberculosis* [141]. In HIV-positive individuals, tuberculosis is among the deadliest contagious diseases, leading to an increase in mortality rates and drug resistance among immunosuppressed individuals. To detect TB disease, researchers developed a theoretical technique for a photonic detector using a 1D-PC of alternating layers. On the performance of the proposed design, the effects of different TB blood concentrations on changing the thickness of the defect layer, the angle of the incident light, and the number of periods were studied. The defect peak was shifted to a lower wavelength region by shifting the concentrations of TB blood samples from normal to infected samples. The sensitivity of the proposed sensor was enhanced by increasing the defect layer thickness and angle of incidence, and the sensitivity reached 1390 nm/RIU with a LoD of 1.5 × 10−6RIU [142]. Yesudasu et al.’s SPR biosensors measure the amount of analyte present in the sample by sensing variation in the refractive index of the medium near the sensor chip surface caused by analyte binding to the immobilized receptor surface. The sensor chip can be reused, which lowers the experimental cost; however, the chips are expensive which makes SPR-based biosensors over-priced. Several researchers have used SPR biosensors for Mtb detection, which are classified based on biological element recognized by them [143]. The table (Table 1) represented below shows portable devices that has the ability to detect diseases accurately through specific markers/biomarkers. 

## 6. Further Developments for the Future in Nanoscale

Some of the most recent research is on smart nanomaterials, which has changed the medical industry. One such smart nanomaterial is theranostic nanovectors; they are made by combining diagnostic and therapeutic capabilities. Using surface-enhanced Raman scattering of such nanovectors used as signature, they are helpful for diagnosis [159]. In another experiment, surface-enhanced Raman spectroscopy as the principal technique and pH sensitive molecular probe 4-mercaptobenzoic acid was used to detect the pH of cancerous and non-cancerous cells [160]. Although much progress has been made in the field of multi-responsive nanomaterials, much more is needed to exploit these materials [161].

Electrical fields are necessary for intercellular communication, muscular contraction, neural signaling, and sensory perception. Mechanical forces are important in cell differentiation, tissue structure, and illnesses including cancer and heart disease. Estimating the forces as well as fields of a biological system is therefore critical for understanding physiology and disease pathology, as well as designing medicinal treatments for repair and recovery. For DNA stretching and Brownian motion, optical tweezers are used, while plasmonic and FRET (Forster resonance energy transfer) are used to estimate protein unfolding and hydrogen bonding. Atomic force microscopy is used to quantify hydrogen and covalent bonds cellular contraction as well as protein unfolding. Using quantum dots stress induced by cellular contraction or covalent bonds present induces crystal field shifts. Laser ablation is used to measure recoil velocity [162]. However, all of these devices need more focus, and much more is required to arrive at complete commercialization for such devices. Therefore, much more research is required to bring them to actual use.

## 7. Summary and Conclusions

In our review, we have studied various smart devices such as wearable devices, implantable devices, ingestible devices, and portable devices. Further under portable devices, we learned about portable devices for health monitoring, as well as portable devices for infectious and non-infectious diseases. From this study, we have learned that these devices are extremely useful in nature since they are faster to respond and are highly specific. They are made up of different kinds of biosensors and/or sensors, whether it may be piezoelectric, capacitive, piezoresistive, MEMS-based, optical, etc. These devices are extremely useful in detecting diseases, and they are very important in early detection, which many of the conventional devices fails to achieve.

Different wearable devices such as skin patches and contact lenses are useful for blood pressure monitoring, heart rate, and body temperature monitoring. Furthermore, they can also be used for quantifying different body fluids such as glucose, lactose, etc., as well as the detecting amount of OH^−^, H^+^, Cu^2+^, Fe^2+^, etc. present in the body. Implantable devices have evolved over the ages starting from external cardiac pacemakers to implantable cardioverter defibrillators. Such devices have neurostimulators that are attached to the collarbone and their configuration can be altered according to the need of the patient. They are efficient in preventing sudden cardiac deaths. With a similar mechanism, deep brain stimulation is being set up that can be used with electrodes inserted into different areas of the brain depending on the type of impairment which is being faced by the patient. Such use can be helpful for correcting Parkinson’s disease, Alzheimer’s, and depressive disorders. Most importantly, such procedures are reversible with minimal side effects and are minimally invasive in nature relative to other procedures. Ingestible devices are ingestible smart pills that can be useful for monitoring the gut and its composition; they can also monitor different internal body temperature and can be used for capsule endoscopy. Such devices are also useful for monitoring GERD (gastroesophageal reflux disease) as well as other conditions.

Portable devices consist of different sensors/biosensors that detect or monitor several infectious and non-infectious diseases. They can be also used for health monitoring by using head-mounted devices, smart watches, e-textiles, electronic shoes, etc., which can provide real time data in a non-invasive manner. The health monitoring devices can be used for monitoring different body constituents and can be used for monitoring temperature, heart rate, blood pressure, etc. Electronic shoes are used for reducing tremors and can be a help for patients with Parkinson’s disease. Portable devices used for detecting non-infectious diseases may involve various cancers such as ovarian cancer, colorectal cancer, and so on. They can be detected with the presence of analytes. Such sensors are also known to be biosensors, which are also useful for detecting different infectious diseases such as AIDS, diseases caused by human cytomegalovirus, COVID-19, Zika virus, etc. Such biosensors maybe be based on genosensors, immunosensors, aptasensors, potentiometric sensors, electrochemical DNA sensors, optical sensors, FET-based sensors, chip NMR sensors, on-chip flow cytometry, impedance spectroscopy, mass-based biosensors, colorimetric biosensors, conductometric sensors, fiber optic evanescent wave biosensors, etc.

Further call for research and development for such devices will not only help to increase knowledge but will help humanity as a whole. Thus, such miniaturized smart devices are important in these days of IoTs and many of them can be easily integrated with a smart phone, laptop, or other interfaces, which makes them important for real-time monitoring, thus helping both the patient and the doctor to achieve a broader perspective.

## Figures and Tables

**Table 1:** Table representing portable devices and their use in infectious and non-infectious diseases.

Portable Device/Biosensor	Disease	Causative Agent	Principle	Disease Type/Marker	Limit of Detection	Advantages	Disadvantages	Reference(s)
Amperometric	New castle disease	Paramyxovirus	Enzyme label immunoassay	Infectious; Antigen of New Castle Disease	11.1 ng mL^−1^	Short time to detect	Solution contamination	[144]
Amperometric	Forest spring encephalitis	Tick-borne encephalitis virus	Sandwich gold-labelled immunoassay	Infectious; Antigen and Protein A	0.0000001 mg mL^−1^	Can be detected in a wide concentration range	Unstable substrates may limit use of such sensors	[145]
Amperometric	Japanese b encephalitis	Japanese encephalitis virus	Probe and label-free immunoassay	Infectious; Fe^2+/3+^ probe	0.000000006 lg pfu mL^−1^	Quick plaque formation	-	[146]
Geno- and immunosensors	Zika fever	Zika virus	(Genosensor) isothermal amplification of viral RNA via nucleic acid sequence-based amplification. (Immunosensor) IDE (interdigitated electrodes) Gold array	Infectious; Zika RNA Zika Protein	3 fM 10 pM	Short time to detect Highly specific Low cost	Elapsed time	[120]
Potentiometric	Hepatitis B	Hepatitis B virus	Enzyme label immunoassay	Infectious; Enzyme labeled with horse radish peroxidase	50 fM (approx)	Rapid detection High stability Highly sensitive	Extremely dependent on polymerization Controlled conditions are required	[147]
Light Adressable Potentiometric (LAPS)	New castle disease	Paramyxovirus	Sandwich enzyme-label immunoassay; Field effect transistor technology	Infectious; N type silicon doped with phosphorus	2 ng mL^−1^	Rapid detection Highly sensitiveResponds on a wide range	Variation of sensitivity	[148]
Light Adressable Potentiometric (LAPS)	Venezuelan equine encephalitis	Venezuelan equine encephalitis virus	Sandwich enzyme-label immunoassay; Field effect transistor technology	Infectious; N type silicon doped with phosphorus, immunofiltration enzyme assay in conjugation with LAPS	30 ng mL^−1^	Rapid detection Highly sensitive Responds on a wide range	-	[149]
Impedance Spectroscopy	Hepatitis B	Hepatitis B virus	Immunoassay	Infectious; Before and after antigen–antibody contact, the electron transfer resistance of a redox probe varies.	8 ng mL^−1^	Rapid detection Highly sensitive Responds on a wide range	On absence of Au nanoparticle and PVB, it retained sensitivity of 27.6%. Antigen and serum may interfere to cause inhibition impedance	[150]
Conductometric	Bovine viral diarrhea	Pestivirus	Sandwich immunoassay	Infectious; Antigen–antibody interaction using conducting polyalanine label	100–10,000 CCID mL^−1^	Responds on a wide range Reaction occurs in less than a minute	Monoclonal antibodies are significantly more susceptible to epitope loss as a result of chemical treatment	[151]
Fiber optic evanescent wave biosensor	New castle disease	Paramyxovirus	Sandwich immunoassay with fluorescein labeling	Infectious; Polyclonal antibody covalently attached to an aminosilane-coated quartz fiber, fluorescein-labeled anti-ND used to identify it	10 ng mL^−1^	Sample analysis can be done along long distances Rapid detection Responds on wide range	-	[152]
Electrochemical Immunosensor	Ovarian cancer	-Type- I-genome alterations in KRAS, BRAF, PTEN, PIK3CA, ARID1A. -Type- II- TP53 mutations	Sandwich-based method conjugation of nanoparticles and antigen	Non-infectious; CA-125 tumor marker	0.0016 U/mL	Highly specific Proteins other than CA-125 showed no interference on high sensitivity or high specificity.	-	[153]
Colorimetric Biosensor	Ovarian cancer	-Type- I-genome alterations in KRAS, BRAF, PTEN, PIK3CA, ARID1A. -Type- II- TP53 mutations	Electric field approach; biotin doped polypyrrole immunosensor based on colorimetric methods	Non-infectious; CA-125, PSA, CEA	PSA-0.7 pg/mL CA-125-0.0005 U/mL CEA-0.8 pg/mL	Rapid detection Direct detection	-	[154]
Mass-Based Biosensor	Ovarian cancer	-Type- I-genome alterations in KRAS, BRAF, PTEN, PIK3CA, ARID1A. -Type- II- TP53 mutations	Based on gold nano material	Non-infectious; CEA	2.5 pg/mL	Highly specific Precise analyte binding	-	[155]
Optical Biosensor	Ovarian cancer	-Type- I-genome alterations in KRAS, BRAF, PTEN, PIK3CA, ARID1A. -Type- II- TP53 mutations	Biochip-based assay	Non-infectious;CA-125	-	10–100 times sensitive Early detection Uses Aptamers which is more stable and versatile than antibodies.		[156]
(Portable Electronic Nose) PEN 3 (E-Nose)	Colorectal cancer	TP53, KRAS, BRAF, and MMR gene Alleles mutation, 18qLOH, CpG methylation	Metal oxide-based detection and analysis through neural network and random forest	Non-infectious; Aromatic, aliphatic, hydrogen, methane, sulfur as substrates for sensing	-	Highly sensitive	Less specificity Cross sensitivity to possible inorganic gases	[17]
GC-TOF-MS (Gas Chromatography-Time of Flight-Mass Spectrometry)	Colorectal cancer	TP53, KRAS, BRAF and MMR gene alleles mutation, 18qLOH, CpG methylation	Volatile organic compound-based detection and analysis through neural network and random forest	Non-infectious; Different volatile organic compounds used as markers for quantification	-	Highly sensitive Highly specific	Detects molecules, having more than 3 carbon atoms only	[17]
COVID-19 FET Sensor	COVID-19	SARS-Cov2 virus	Field effect transistor	Infectious; Antibody conjugated graphene	1 fg/mL	Highly sensitive Rapid and real-time Highly specific	-	[157]
RT-LAMP NBS	COVID-19	SARS-Cov2 virus	Reverse transcription loop mediate isothermal amplification; F1ab and nucleoproteins-based nano biosensor	Infectious; F1 ab and nucleoproteins	12 copies	100% sensitivity 100% specificity Takes less time	-	[116]
Electrochemical DNA Sensor	HCMV Associated disease	Human cytomegalo virus	Based on an EPAD that includes Zn–Ag nanoblooms	Infectious; HHV-5 DNA	97 copies per mL	Quick fabrication technique can be used to develop it	Expensive wax printers are required After wax deposition, a further heating step is required	[140]
Optical Biosensor	HCMV Associated disease	Human cytomegalo virus	SPR-dependent method	Infectious; Protease and peptidase reaction of HCMV	-	Reusable Label free High sensitivity	Low selectivity Nonspecific binding	[139]
Piezoelectric Biosensor	HCMV Associated disease	Human cytomegalo virus	The technique of strand displacement amplification was used	Infectious; HCMV Nucleic acid	-	Takes less time to detect Highly sensitive Real time detection	High temperature sensitivity Inability to amplify long sequences	[158]
On Chip Flow cytometry	AIDS	HIV	Flow cytometry	Infectious; CD4^+^ detection	10 µL (whole blood required to detect)	Label-free detection Lens-less imaging	Difficult for clinical use unless modified with sheath-less focusing techniques	[16]
Chip NMR Biosensor	Tuberculosis	Mycobacterium Tuberculosis	Miniaturized diagnostic magnetic resonance	Infectious; Mycobacterium detection from sputum	1 ng (approx.)	High Sensitivity and specificity	Micro-coil resistance Signal detecting circuitry is monolithically integrated on a single integrated circuit chip	[134]

## Data Availability

Not applicable.
